# A Turf-Based Feature Selection Technique for Predicting Factors Affecting Human Health during Pandemic

**DOI:** 10.3390/life12091367

**Published:** 2022-09-01

**Authors:** Alqahtani Saeed, Maryam Zaffar, Mohammed Ali Abbas, Khurrum Shehzad Quraishi, Abdullah Shahrose, Muhammad Irfan, Mohammed Ayed Huneif, Alqahtani Abdulwahab, Sharifa Khalid Alduraibi, Fahad Alshehri, Alaa Khalid Alduraibi, Ziyad Almushayti

**Affiliations:** 1 Department of Surgery, Faculty of Medicine, Najran University, Najran 61441, Saudi Arabia; 2 Faculty of Computer Sciences, IBADAT International University, Islamabad 44000, Pakistan; 3 Department of Chemical Engineering, Pakistan Institute of Engineering and Applied Sciences (PIEAS), Islamabad 44000, Pakistan; 4 Department of Computer Science, HITEC University, Taxila 47080, Pakistan; 5 Electrical Engineering Department, College of Engineering, Najran University Saudi Arabia, Najran 61441, Saudi Arabia; 6 Department of Pediatrics, College of Medicine, Najran University, Najran 61441, Saudi Arabia; 7 Department of Radiology, College of Medicine, Qassim University, Buraidah 52571, Saudi Arabia

**Keywords:** mental stress, COVID-19, feature selection, artificial intelligence, human health, pandemic, lock down

## Abstract

Worldwide, COVID-19 is a highly contagious epidemic that has affected various fields. Using Artificial Intelligence (AI) and particular feature selection approaches, this study evaluates the aspects affecting the health of students throughout the COVID-19 lockdown time. The research presented in this paper plays a vital role in indicating the factor affecting the health of students during the lockdown in the COVID-19 pandemic. The research presented in this article investigates COVID-19’s impact on student health using feature selections. The Filter feature selection technique is used in the presented work to statistically analyze all the features in the dataset, and for better accuracy. ReliefF (TuRF) filter feature selection is tuned and utilized in such a way that it helps to identify the factors affecting students’ health from a benchmark dataset of students studying during COVID-19. Random Forest (RF), Gradient Boosted Decision Trees (GBDT), Support Vector Machine (SVM), and 2- layer Neural Network (NN), helps in identifying the most critical indicators for rapid intervention. Results of the approach presented in the paper identified that the students who maintained their weight and kept themselves busy in health activities in the pandemic, such student’s remained healthy through this pandemic and study from home in a positive manner. The results suggest that the 2- layer NN machine-learning algorithm showed better accuracy (90%) to predict the factors affecting on health issues of students during COVID-19 lockdown time.

## 1. Introduction

The COVID-19 was triggered by Sars-Cov-2 coronavirus, which was initially identified in Wuhan, China in December 2019 [1,2]. This disease spread in the whole world rapidly and has significantly affected many aspects of life including mental health, social life, supply chain, energy consumption, education, etc., [3,4]. Lockdown measures were taken by governments all over the world to impede the disperse of the disease. People all over the world were restricted to quarantine and keep social distancing to determine the number of people who have become infected [5]. Studies have shown that the lockdown during this COVID-19 had different physiological effects including anxiety, stress, confusion [6], and anger [7]. Similar effects were observed in the education domain and various educational stakeholders were affected by lockdown in COVID-19. According to a report of UNSECO [8], about 1.6 billion students faced school closure issues. Face-to-face education was replaced by e-learning. This transformed the lives of the students reducing them to their homes. Students’ mental and physical health is affected by the COVID-19 lockout situation. Different studies are taking part to figure out the reasons causing the disturbance in the students’ mental health. During COVID-19, a variety of statistical tools were used to investigate the elements that influence the students’ mental health. The results of different existing studies reported that lockdown causes depression, anxiety, mental stress and health issues in quarantined populations during COVID-19 [9]. Furthermore, social distancing and different lockdown measures during COVID-19 negatively affect the health of student [10]. The efficient execution of education depends on the health of students. As the students are the main pillar of society and the nation’s leadership and control will rely on them in the future. Therefore, it is necessary to put maximum possible effort to maintain the health of students [11].

Artificial intelligence (AI) plays a vital role in predicting coronavirus effects in the future by analyzing the covid data [12]. Different Al-based supervised and unsupervised algorithms are being employed in studies for COVID-19 predictions and analysis [13]. As the education sector is the base of every country’s development, so AI techniques help the educational stakeholders and government officials of countries all over the world to plan strategies and techniques to maintain the health of students. The focus of this study is to employ the AI-based technique for the identification of factors affecting the students’ mental health in the pandemic of COVID-19. The following are the primary contributions of the proposed work:

- Identifying the factors affecting the health of students in the lockdown phase of COVID-19;
- To assist the educational stakeholders in taking proactive measures for maintaining the student’s health for the duration of COVID-19;
- Proposing AI-based identification of features affecting the student’s health during the lockdown period of COVID-19;
- Explore AI approaches namely RF, GBDT, SVM, and NN along with Turf feature selection for selecting the optimal feature set affecting the health of students.

In this study, COVID-19 related student data will be evaluated in order to determine factors affecting students’ health during COVID-19 lockdown. The paper is structured in the subsequent pattern: a summary of related literature is provided in Section 2; the suggested AI-based strategy is described in detail in Section 3; Section 4 presents the analysis evaluation of the proposed technique; and the research and future work is summarized in Section 5.

## 2. Related Work

Different studies are conducted to illustrate the effects of lockdown during COVID-19. In this section, an overview of existing approaches is presented, focusing on the students’ mental health during the lockdown period in COVID-19. Different studies in different countries are conducted all over the world to analyze the health of students during the lockdown in this pandemic situation. Some of the studies are selected from the related work on the mental and physical health of the students, available on Google Scholar. Table 1 shows the reference of the papers, country in which the study was conducted, also presents the different variations in sizes of datasets collected for analyzing the factors affecting the health of students in a pandemic situation. Furthermore, Table 1 analyzes that whether the existing studies are utilizing AI techniques or not. In the end, Table 1 also presents what is the conclusion of the recent studies regarding the factors affecting the mental health of students.

The paper presents 16 most relevant literature on student’s health in COVID-19. Recent studies are evaluated on 5 different parameters: the country through which dataset is taken, study level of student’s understudy, number of students in the dataset, utilization of machine learning technique for identification of factors affecting student’s health, and, lastly, the factor identified by the existing studies that may affect the health of students all through lockdown phase of COVID-19. Different the different levels and sizes of students with varying datasets sizes. The studies focus on graduate, undergraduate, college, public schools, and medical and forestry students of different countries. The recent literature indicates that there is so much gap in studies regarding machine learning utilization for analyzing the mental health of students. Different factors come across while analyzing the existing literature on the students’ mental health. Mainly, the following factors were found to be very crucial in association with the students’ mental health in the COVID-19 pandemic.

- Loneliness [6];
- The feeling of isolation [13];
- Fear of academic year loss;
- Availability of space for studies;
- Family functioning;
- Females have more mental health issues than male students during COVID-19;
- Fear of own health;
- Fear of dear one’s health;
- Poverty;
- Student Counseling.

Different factors are found in the literature that has an association with the mental health of students. These factors will help the educational admiration to take measures for maintaining the health of students during COVID-19. Different remote techniques and activities should be planned by educational stakeholders to minimize the anxiety of students during the lockdown period of the pandemic. However, as the health of students is an important concern so there is a need for deep insight into the data of students during such pandemic situations. However, the need for AI algorithms is still there for a better insight into data and its analysis. Main shortcomings in recent studies regarding the health of students in COVID-19 are still required to address, some of the shortcomings found in the literature that may help the educational stakeholders to build educational strategies. Firstly, there is a need to the utilization of feature selection techniques to identify the features affection health of students during the lockdown in COVID-19. To our knowledge, there has never been a study that conducted a comprehensive literature analysis and identified factors affecting the health of kids during COVID-19’s lockdown period based on feature selection, whereas [28] has presented and utilized AI, but did not consider feature selection. In the coming sections of this article, we will discuss our novel proposed approach for the analysis of factors affecting the health of students in COVID-19.

## 3. Methods and Materials

**Figure 1:**
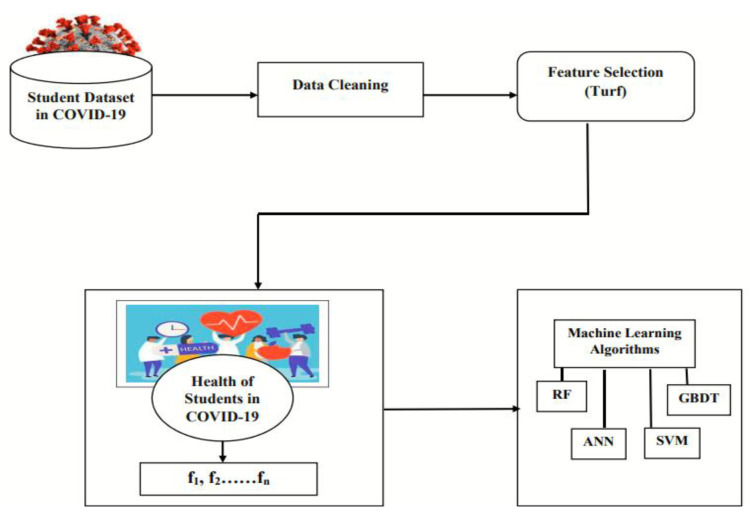
Proposed flow AI-Based feature selection of factors affecting the health of students in COVID-19.

In this section proposed approach for identifying the factors affecting the health of students is presented. As it is very important to figure out that what are factors affecting the health of Figure 1 presents the main flow of the proposed approach main steps of the proposed approach is as follows:

- Dataset Selection;
- Dataset Cleaning;
- Feature Selection;
- Machine learning algorithm.

Each of the steps is explained further in detail in the coming subsections.

### 3.1. Student Dataset in COVID-19

A benchmark dataset of 1182 students in COVID-19 [29] is utilized to analyze the factors affecting the health of students in the lockdown period of COVID-19. The dataset is freely available and, hence, utilized easily for research purpose. Table 2 describes the main properties of the student dataset.

### 3.2. Data Preprocessing

Python programming language platform is utilized for coding the proposed approach, and its various libraries like NumPy, pandas for better insight of data [30]. Different steps are taken to preprocess the imbalanced dataset, firstly by scaling and data cleaning by deleting ids, dropping duplicating rows, and filling all NA values. Moreover, categorical features are mapped to numbers. Furthermore, to convert the text features like (stress buster, what you miss most), pretrained bert is utilized for generating word vectors. Then words are mapped to a single feature by following the normalization formula as:

(1) $$x=\frac{sum\left(vector\right)}{max\left(vector\right)-min\left(vector\right)}.\;$$

**Figure 2:**
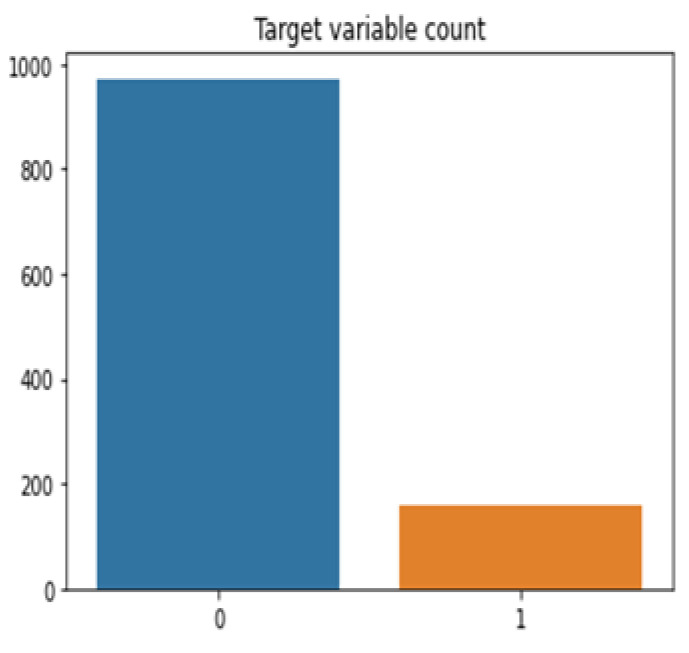
Variable count before sampling.

**Figure 3:**
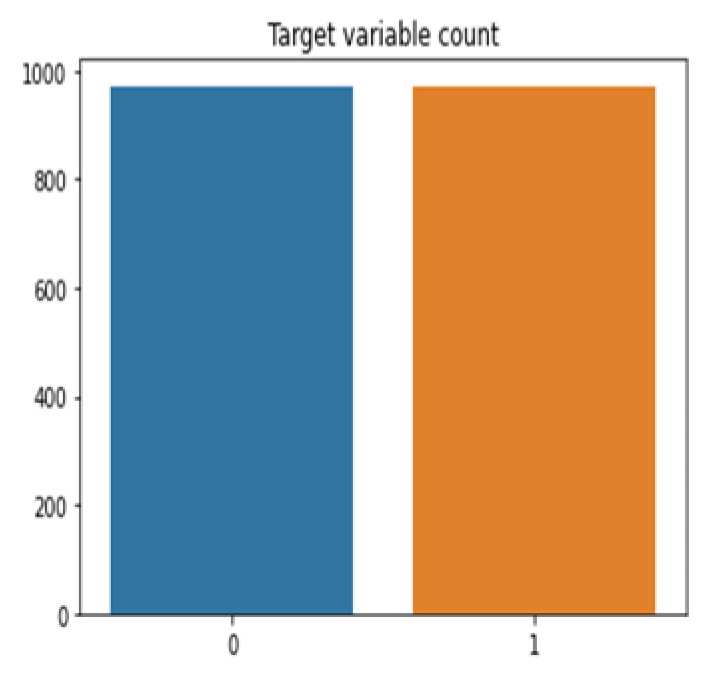
Variable count after sampling.

Figure 2 and Figure 3 represents variable count after and before sampling, whereas SMOTE (Synthetic minority oversampling technique) addresses imbalance class issues very effectively in various domains of research [31]. SMOTE oversampling technique is applied to resample student’s datasets for COVID-19. Based on feature space similarity, the SMOTE approach combines extra minority samples [32]. Let *k* = nearest neighbor for xi using Euclidean distance.

Random Selection of *k* nearest neighbor

Feature vector difference between *k* and *x_i_*

Adding M in *x_i_*

Equation (2) presents the formula for calculating SMOTE

This is example 2 of an equation:

(2) $$x_{new\;}=x_{i}\left(x_{i}^{k}-x_{i}\right)\times \delta .\;$$

$x_{i}^{k}$ = A nearest neighbors of *x_i_*, and δ is an arbitrary value belongs to (0, 1).

### 3.3. Feature Selection

Feature selection is a process to obtain an optimal set of features, to obtain better classification accuracy. There are different types of feature selection algorithm filter and wrapper feature selection. Filter feature selection is high in speed [33] and consumes less time, and is the main reason for selecting filter feature selection in our proposed approach. Filter feature selection is further divided into two types, univariant and multivariant filter feature selection methods. The univariant filter feature ignores the features dependencies and that leads to a poor selection of feature set [34], whereas multivariant feature selection takes consideration of feature dependencies while selecting the feature set [35]. Turf is the tuned form of Relief multivariant filter feature selection. When selecting relief features, feature dependencies are taken utilizing the full feature vector, which may ignore the noisy features, so that Turf feature selection step by step low-quality features, hence, generating optimal feature set [36]. The Turf algorithm is presented in Algorithm 1.
**Algorithm 1.** TuRF algorithm [36].a = features in datasetLet p = iterationsFor                   i:= 1 to p doEstimation of feature weights through ReliefFFeatures sorting through weight+ +   remove p/a of outstanding features with smallest weightsend forreturn                  final ReliefF weight estimations for outstanding features

### 3.4. Machine Learning Algorithms

After the selection of features, classification is performed. SVM (Support Vector Machine) is a classifier for binary classification of data. The hyperplane is used to solve the learning problem in SVM. A robust method with different kernel values is considered one of the best classifiers for classification [37]. RF (Random Forest) utilized various trees to predict. It is being utilized by different research areas of research with remarkable results. RF produces high classification accuracy with an even dataset with a large number of features. It handles unbalanced data by accessing important features. Whereas GBDT (Gradient Boosting Decision Tree) is selected due to its property of selecting fewer parameters as compared to the other classification algorithms. In existing research, in machine learning, GBDT shows tremendous results. It is based on the CART algorithm. GBDT merges the concept of regression and boosting tree and intends the use of residual gradient to optimize the assimilation process of regression tree [38]. ANN (Artificial Neural Network) is a popular classification technique utilized in different areas of research like agriculture, medical, security, education, business, art, etc. It is very easy to use and can manage complex data [39]. Moreover, the performance of the proposed approach presented in this paper is evaluated through accuracy, precision, recall, and f-measure, whereas accuracy is defined as the predicted observations over a total number of observations [40,41,42]. Precision is the fraction of the recovered instances that belong to the target class, whereas F-measure is the harmonic mean of precision and recall. Equations (3)–(6) presents the formula of evaluation parameters, whereas *TP*, *FN*, and *FP* stand for true positive, false negative, and false-positive respectively.

(3) $$\mathrm{Accuracy}=\frac{TP+FN}{TP+FN+FP+FN}$$

(4) $$\mathrm{Precision}=\frac{TP}{TP+FP}$$

(5) $$\mathrm{Recall}=\frac{TP}{TP+FN}$$

(6) $$F-\mathrm{Measure}=\frac{2\left(Precision\times Recall\right)}{Precision+Recall}$$

whereas Table 3 presents the Parameters of classification algorithm utilized in proposed work.

## 4. Results

**Figure 4:**
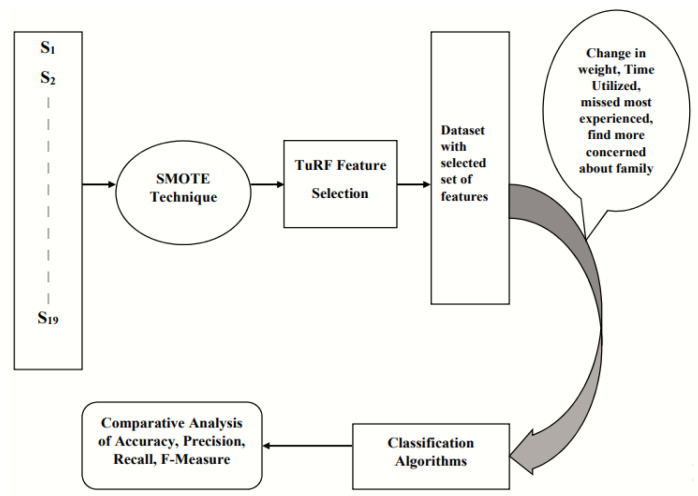
Some feature selection process of factors affecting the student’s health during COVID-19.

Results of the proposed approach for the identification of factors affecting the health of students in COVID-19 will be discussed in detail in this section. Figure 4 explains the proposed method in detail with results. The results show that the dataset of a feature vector of 16 features is balanced through applying SMOTE technique. The health of students is taken as a target feature, and the Turf feature selection technique is utilized to detect the factors influencing the health of students. Different classification algorithms are applied to the selected feature datasets of student’s health during COVID-19. The performance of the suggested method was assessed using accuracy, precision, recall, and f-measure assessment metrics.

**Figure 5:**
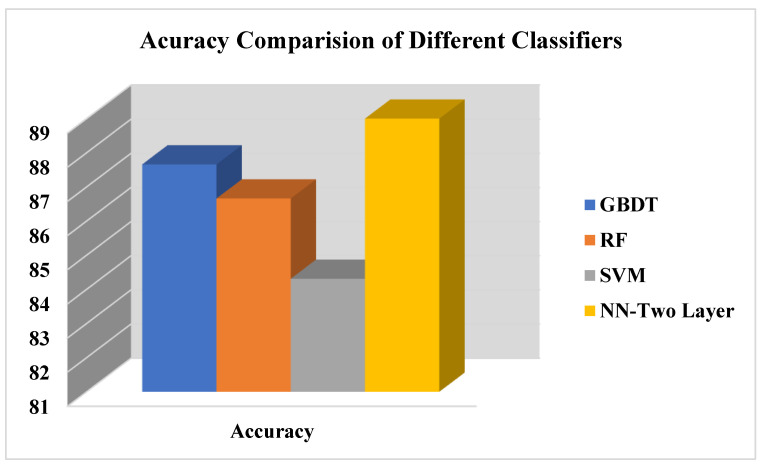
Comparison of accuracy of proposed COVID-19 approach.

The result s shows that the student who utilized their time during lockdown period in COVID-19 in different activities remain healthy. Utilization of time appears as the main factor affecting the health of students. The academic organization may keep that factor in front and must plan activities, guide, and motivate students to participate in some indoor actives in such a way that maintains their health. Emotional attachment of students with family members also affects the health of students, as the fear of any family loss due to COVID-19 affects the health of students. Moreover, change in the weight of students during COVID-19 also affects the health of students. Figure 5 presents the results of four classifiers, GBDT, RF, SVM, and NN, on students COVID-19 dataset, whereas the accuracy describes the number of healthy students correctly classified by proposed work over a total number of students. Results show that a Neural network (NN) outperforms other existing classification algorithms in terms of accuracy. However, GBDT also performs well on students COVID-19 dataset and showed around 87% of accuracy. Equation (7) presents the accuracy formula for the student COVID-19 dataset.

(7) $$Accuracy=\frac{Number\;ofstudentscorrecly\;classified\;}{Total\;number\;of\;students}$$

**Figure 6:**
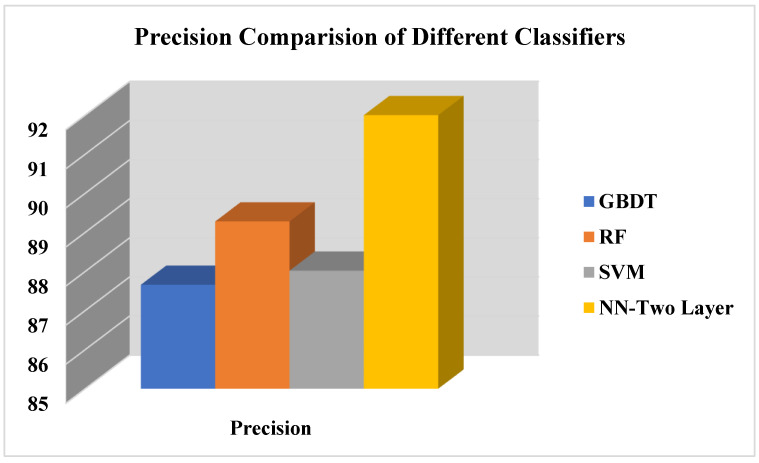
Comparison of precision of proposed COVID-19 approach.

Figure 6 presents the performance evaluation of the proposed work in terms of precision, whereas precision calculates the number of healthy students in the COVID-19 student dataset correctly classified by proposed work divided by the total number of healthy students in the COVID-19 dataset, classified by the proposed approach. Results show that neural network performs better than other classification algorithms. Equation (8) presents the formula of precision for calculation precision of proposed approach on student COVID-19 dataset.

(8) $$Precision=\frac{Number\;of\;healthy\;students\;identified\;by\;propsed\;approach}{Total\;number\;of\;health\;and\;unhealthy\;students\;\;classified\;by\;proposed\;approach}$$

**Figure 7:**
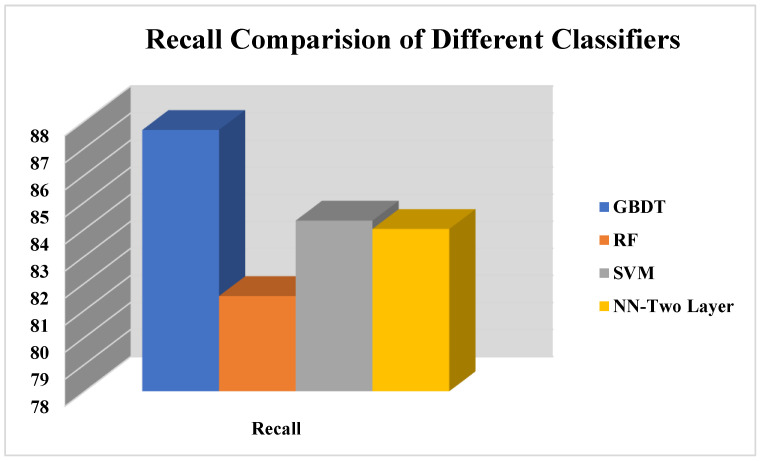
This Comparison of recall of proposed COVID-19 approach.

The results in Figure 7 show the performance evaluation of the proposed work in terms of recall. The recall is the calculation of a total number of healthy students in the COVID-19 student dataset classified by the proposed approach divided by the total number of healthy students in the COVID-19 student dataset. The results show that the GBDT classifier outperforms other classifiers in recall performance evaluation measures. Furthermore, RF and NN also show better performance. Equation (9) presents the formula for calculating the recall for evaluating proposed approach on students COVID-19 students.

This is example 2 of an equation:

(9) $$Recall=\frac{Number\;of\;healthy\;studentsclassfied\;by\;the\;proposed\;approach}{Total\;number\;of\;healthy\;students}$$

**Figure 8:**
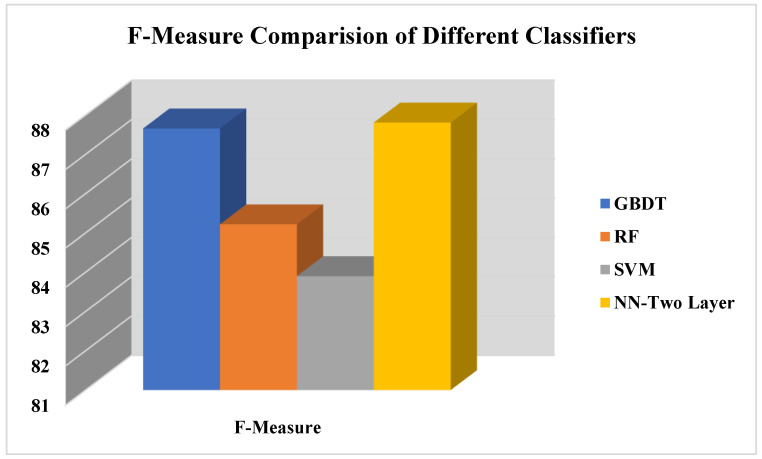
This Comparison of f-measure of proposed COVID-19 approach.

Figure 8 presents a comparison of the performance of four classifiers in terms F-measure performance evaluation measure, whereas the f-measure of the proposed approach considers precision and recall both, presented already in Equation (6). Results show that GBDT and NN give better performance on the proposed work on the COVID-19 student dataset in terms of F-measure.

## 5. Conclusions

COVID-19 affects every field of life, the educational sector all over the world faces different issues. During the lockdown, students face a lot of issues, whereas health issue becomes the main issue. Results presented in the proposed approach identifies the main factors affecting the health of students during the lockdown. Results show that the health of students affects the factors that how they utilized their time during the lockdown in COVID-19, whereas weight and family concerns also appear as factors affecting the health of students during a lockdown of COVID-19. Henceforth, there is a need to take proactive measures to discover the approaches to sustain the health of students, either by guiding them in health time utilization activities or by counseling them about family matters. These well-timed taken measures may reduce the health issues in students caused by pandemic situation in COVID-19. Moreover, reported results in this paper `show that neural network outperforms and shows 90% accuracy on the proposed approach as compared to GBDT, RF, and SVM.

## Figures and Tables

**Table 1:** Analysis of studies on student’s health during COVID-19 in different countries.

Reference	Country	Level of Students	Size of Dataset	Machine Learning	Effect on Mental and Physical Health in COVID-19
[6]	Malaysia	Postgraduate	-	No	Loneliness, anxiety, stress, and depression.
[14]	Asian students in Poland	Medical students	85	No	Feeling of isolation to students who live abroad.
[15]	Bangladesh	College Students	400	No	In COVID-19, perceptions of e-Learning failure and fear about academic year failure were connected with psychological distress.
[16]	Bulgaria	Graduate and undergraduate students	134	Yes	Availability of separate rooms for students affects their education.
[17]	China	Non-graduating undergraduate students	1172	YES XGBOOST	School closure, Social distancing or Isolation, and Online learning are the reason for anxiety.
[18]	China	Secondary vocational students	5783	No	Good family functioning can positively affect the mental health of students.
[19]	Philippines	College Students	952	No	Socioeconomic gaps and the digital divide affect the mental health of students.
[20]	India	Undergraduate and post-graduate	516	Yes	Uncertainty regarding examination affects the mental health of students.
[21]	Jordan	Medical Student	1404	No	Students focus on strategies to prevent covid.
[22]	Pakistan	Higher Educational Institutions	494	No	Unaffordability of digital devices and the internet.
[23]	USA (United States of America)	University Students	195	No	Fear of own health and dear one’s health affects the mental health of students.
[24]	UAE (United Arab Emirates)	Medical and non-medical students	1485	No	Fear of the unknown might affect the mental health of students so that students must be aware of the COVID-19.
[25]	Saudi Arabia	University students	400	Yes	Females and fourth-semester students face anxiety during COVID-19.
[26]	New Zealand	Mater level Graduate-level Teaching degree (Mathematics education learning)	3	No	Teachers help in the transition of a new way of learning that affects students.
[27]	Greece	Undergraduate forestry students	181	No	Students must be counseled properly to control negative emotions during the lockdown.
[7]	Iran	Public school students	20,697	No	Behavioral and socializing changes during COVID-19 affects mental health.

**Table 2:** Student COVID-19 dataset description.

Number of Students	1182
Number of features	19
Features	Id of the student, home location of students, Student_age, Time consumed _online Class, Rating of Online Class experience, Instruction medium for an online class, Time consumed_ self-study, Time consumed_ fitness, sleeping_ time, Time consumed_ social media, preferred social media platform, Time consumed_ TV, meals _per day, changes _weight, Health issue_ lockdown, Stressbusters, Utilization _time, what you miss the most
Target feature	Health issue during lockdown
Number of classes	2

**Table 3:** Parameters of classification algorithm utilized in proposed work.

SR	Name	Parameters
1	GBDT	n_estimators = 19, learning_rate = 0.3, max_depth = 7, random_state = 0
2	SVM	SVC(C = 5, break_ties = False, cache_size = 200, class_weight = Balanced, degree = 3, gamma = 11, kernel = ‘rbf’)
3	Random Forest	bootstrap = True, criterion = ‘gini’, max_depth = 15, max_features = ‘auto’, min_samples_leaf = 1, min_samples_split = 2, n_estimators = 20
4	NN-2 Layers	Momentum0.9, learning rate = 0.003, layers = 2, drop_out = 0.1, optimizer = adam, Loss Binary Class
